# Identification and Validation of Hub Genes Associated With Hepatocellular Carcinoma *Via* Integrated Bioinformatics Analysis

**DOI:** 10.3389/fonc.2021.614531

**Published:** 2021-06-30

**Authors:** Sumei Wang, Zuoli Song, Bing Tan, Jinjuan Zhang, Jiandong Zhang, Shuye Liu

**Affiliations:** ^1^ Department of Clinical Laboratory, The Third Central Hospital of Tianjin, Tianjin, China; ^2^ Tianjin Key Laboratory of Extracorporeal Life Support for Critical Diseases, Tianjin, China; ^3^ Artificial Cell Engineering Technology Research Center, Tianjin, China; ^4^ Tianjin Institute of Hepatobiliary Disease, Tianjin, China; ^5^ Department of Surgery, Third Central Hospital of Tianjin, Tianjin, China

**Keywords:** hepatocellular carcinoma, weighted gene co-expression network analysis, biomarker, TCGA, GEO

## Abstract

Hepatocellular carcinoma (HCC) is the most common malignant tumor of the liver, with high morbidity and mortality, yet its molecular mechanisms of tumorigenesis are still unclear. In this study, gene expression profile of GSE62232 was downloaded from the Gene Expression Omnibus (GEO). The RNA-seq expression data and relative clinical information were retrieved from the Cancer Genome Atlas (TCGA) database. The datasets were analyzed by differential gene expression analysis and Weighted Gene Co-expression Network Analysis (WGCNA) to obtain the overlapping genes. Then, we performed a functional enrichment analysis to understand the potential biological functions of these co-expression genes. Finally, we constructed the protein-protein interaction (PPI) analysis combined with survival analysis. MARCO, CLEC4M, FCGR2B, LYVE1, TIMD4, STAB2, CFP, CLEC4G, CLEC1B, FCN2, FCN3 and FOXO1 were identified as the candidate hub genes using the CytoHubba plugin of Cytoscape. Based on survival analysis, the lower expression of FCN3 and FOXO1 were associated with worse overall survival (OS) in HCC patients. Furthermore, the expression levels of FCN3 and FOXO1 were validated by the Human Protein Atlas (HPA) database and the qRT-PCR. In summary, our findings contribute new ideas for the precise early diagnosis, clinical treatment and prognosis of HCC in the future.

## Introduction

Hepatocellular carcinoma (HCC) is one of the most common malignant tumors worldwide and the main cause of global cancer-related death (1). The current treatment principle is comprehensive treatment based on surgical treatment. Non-surgical treatment includes local tumor treatment, radiotherapy, chemotherapy and biological treatment. Liver transplantation technology is gradually becoming mature, but it is also mainly suitable for early HCC patients and the prognosis of late patients is poor (2). However, due to the lack of early diagnosis biomarkers, patients are usually in the late clinical stage at the time of diagnosis, and the overall survival (OS) of HCC patients is very poor. Therefore, it is of great significance to identify effective biomarkers and therapeutic targets for the treatment of HCC.

In recent years, bioinformatics analysis of expression profile has been widely and rapidly used to identify novel and more effective potential biomarkers for cancer treatment and patient prognosis (3–7). However, how to transform the microarray information into a better understanding of biology through traditional differential expression analysis is still a major challenge. Weighted Gene Co-expression Network Analysis (WGCNA) is an advanced method used to construct a co-expression module based on similar gene expression patterns and analyze the relationship between modules and specific features. Here, the differential gene expression analysis and WGCNA are combined to enhance the discrimination ability of highly related genes, which can be used as candidate biomarkers.

In this study, the mRNA expression data of HCC from the GEO and TCGA databases were analyzed by differential gene expression analysis and WGCNA to obtain the overlapping genes. Then, we performed a functional enrichment analysis to understand the potential biological functions of these co-expression genes. Finally, we constructed the protein–protein interaction (PPI) analysis combined with survival analysis. These results will help us to understand the etiology and potential molecular mechanism of HCC, and provide new therapeutic targets or biomarkers for HCC.

## Materials and Methods

### Data Sources and Data Processing

**Figure 1:**
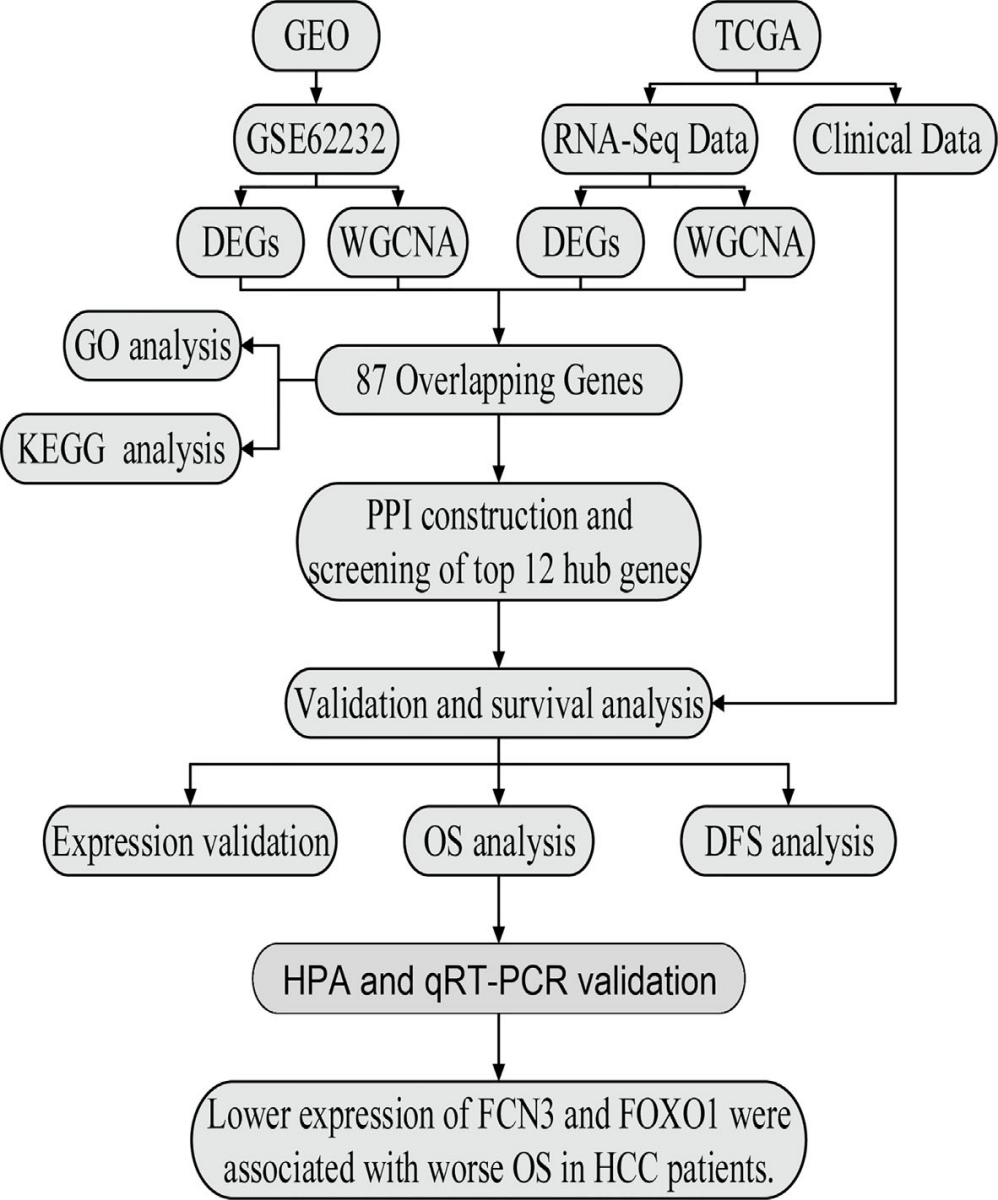
The flow chart of data preparation, processing, analysis, and validation.

The workflow of the analysis is shown in **Figure 1**.

Gene expression profile of GSE62232 (8) was obtained using R package GEOquery from the GEO (http://www.ncbi.nlm.nih.gov/geo/). It was submitted by Sandrine Imbeaud and based on GPL570 platform ([HG-U133_Plus_2] Affymetrix Human Genome U133 Plus 2.0 Array), including 81 HCC samples and 10 normal samples. Probes were converted into gene symbols according to the annotation documents, and repeated probes of the same gene were removed by determining the median expression value of all corresponding probes. Results, 21,654 genes were selected for subsequent analysis. The RNA-seq expression data and relative clinical information of HCC patients were retrieved from the TCGA database (https://portal.gdc.cancer.gov/) (9), including 374 HCC samples and 50 normal samples. In this study, we maintained genes with CPM (count per million) ≥1. After filtering with the RPKM (Reads Per Kilobase per Million mapped reads) function in edge R package (10), 13,913 genes with RPKM value entered our next analysis.

### Differential Gene Expression Analysis

The limma package (Version4.0; http://www.bioconductor.org/packages/release/bioc/html/limma.html) (11) was applied to significance analysis of differentially expressed genes (DEGs) between HCC and normal tissue samples with the following selection criteria: thresholds of |logFC| ≥1.0, and an adjusted p-value of <0.05. The values of genes calculated by limma R package, and genes with expression of <0.5 were deleted. The volcano plot and hierarchical clustering analysis were represented by the R packages “ggplot2” and “pheatmap”, respectively.

### WGCNA and Interaction With the DEGs

WGCNA is an analysis method for analyzing gene expression patterns of multiple samples. It can cluster genes with similar expression patterns and analyze the relationship between modules and specific traits or phenotypes. Co-expression network was established using the WGCNA R package (12) based on the gene expression data profiles of TCGA-HCC and GSE62232.

First, the samples were tested, abnormal samples and genes were eliminated, the Pearson correlation matrix was constructed through the gene correlation coefficient, and the power function apq = |cpq|β (apq = adjacency between gene p and gene q, cpq = Pearson’s correlation between gene p and gene q, and β = soft threshold) was used to convert it into a weighted adjacency matrix. In order to satisfy the scale-free distribution, soft threshold was set as β = 2 and 5. Then we transform the adjacency matrix into topological overlap matrix (TOM). Finally, genes with similar expression patterns were merged into the same module (minimum module size = 50). In order to identify the overlapping genes between important modules and TCGA-HCC and GSE62232 datasets, a Venn diagram was developed using the R package “VennDiagram” (13).

### Function Enrichment Analysis

The clusterProfiler package (14) was used for functional enrichment of Gene Ontology (GO) and Kyoto Encyclopedia of Genes and Genomes (KEGG) (15). Through GO enrichment analysis, we can better understand their biological characteristics. KEGG helps us study the functional interpretation of genes and genomes as a whole network. The GO terms consist of the following three parts: biological process (BP), cell component (CC) and molecular function (MF). Adjusted *p*-value <0.05 was considered to have statistical significance.

### Protein–Protein Interaction (PPI) Network and Hub Gene Identification

The Search Tool for Retrieval of Interacting Genes database (STRING, https://string-db.org/) was used to construct PPI networks (16). According to the confidence score ≥0.4, the genes with significant interaction were screened, and the filtered results were imported into the Cytoscape software 3.8.0 (17) (http://www.cytoscape.org) for network visualization. CytoHubba is used to explore important nodes in biological networks. It provides 11 topological analysis methods, Maximal Clique Centrality (MCC) algorithm was considered to be the most effective method to find the hub nodes (18). Top 12 MCC values were selected and then intersection was taken to get the hub genes in PPI analysis.

### Hub Genes Validation and Survival Analysis

In order to further verify the relationship between hub genes and clinical features, we analyzed the corresponding data from TCGA database for verification. OS was performed by using the survival package in R software and disease-free survival (DFS) analyses of hub genes was analyzed using the online tool Gene Expression Profiling Interactive Analysis (GEPIA) (http://gepia.cancerpku.cn/) (19). The Kaplan–Meier curves were plotted by the expression profiles, which were divided into two groups based on the median expression value of hub genes. For survival analysis, R package “survival” and “survminer” were used to implement log-rank tests, *p*-value <0.05 was considered as statistically significant.

### Validation of Protein Expressions of Hub Genes by the HPA Database

The Human Protein Atlas database (HPA) (https://www.proteinatlas.org/) (20) is dedicated to providing tissue and cellular distribution information of a variety of human proteins. The protein expression of the survival-related genes between HCC and normal tissues was determined using immunohistochemistry (IHC) from the HPA.

### RNA Extraction and qRT-PCR

Total RNA was isolated using the Nuclezol LS RNA Isolation Reagent (ABP Biosciences, Inc). cDNA was synthesized using 1.0 ug of total RNA with the SureScript-First-strand-cDNA-synthesis-kit (GeneCopoeia, Guangzhou). Quantitative PCR was performed for hub genes using the BlazeTaq™ SYBR ^®^ Green qPCR Mix 2.0 kit (GeneCopoeia) with the CFX96 real time quantitative PCR instrument (Bio-Rad, USA). The relative expression levels were determined by the 2^−ΔΔCt^ method and normalized to internal control GAPDH. All qPCR reactions were performed in triplicate. The primers designed by Qingke Biology Co., Ltd are listed as below: FCN3-F: CAGGATGGTTCTGTGGATTT; FCN3-R: TCAGCGTCATAGGTGGTAAA; FOXO1-F: CTTCTGACTCTCCTCCCCACA; FOXO1-R: CCCATCCTACCATAGCCATTG; GAPDH-F: CGCTGAGTACGTCGTGGAGTC; GAPDH-R: GCTGATGATCTTGAGGCTGTTGTC.

## Results

### The Identification of DEGs

**Figure 2:**
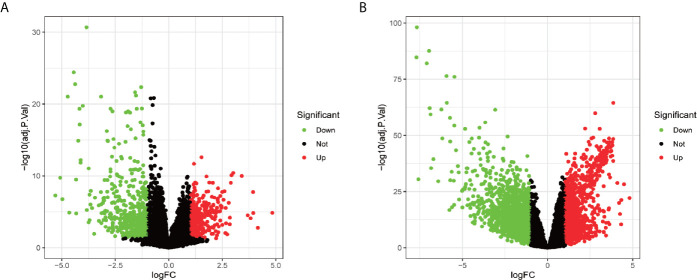
The volcano plot of DEGs in patients with HCC. **(A)** Volcano plot of DEGs in the GSE62232 dataset. **(B)** Volcano plot of DEGs in the TCGA dataset. Up-regulated genes and down-regulated were represented in red dot and green dot respectively.

**Figure 3:**
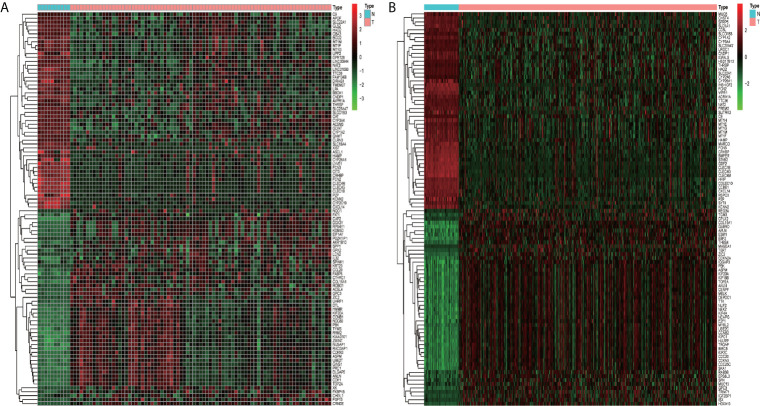
The heatmaps of DEGs between HCC and normal tissues. **(A)** Heatmap of the GSE62232 dataset. **(B)** Heatmap of the TCGA dataset. Red represents the upregulated genes and green represents the downregulated genes.

In total, 1,019 DEGs in the GSE62232 dataset (**Figure 2A**) and 2,703 DEGs in the TCGA dataset (**Figure 2B**) were found to be dysregulated in tumor tissues by the limma package, according to the adjusted *p*-value of <0.05 and a |logFC| ≥1.0. The heatmaps of the two datasets, as shown in **Figures 3A, B**, respectively show that these DEGs can distinguish HCC from normal specimens well.

### Construction of Weighted Gene Co-Expression Modules and Identification of Overlapping Genes With DEGs

**Figure 4:**
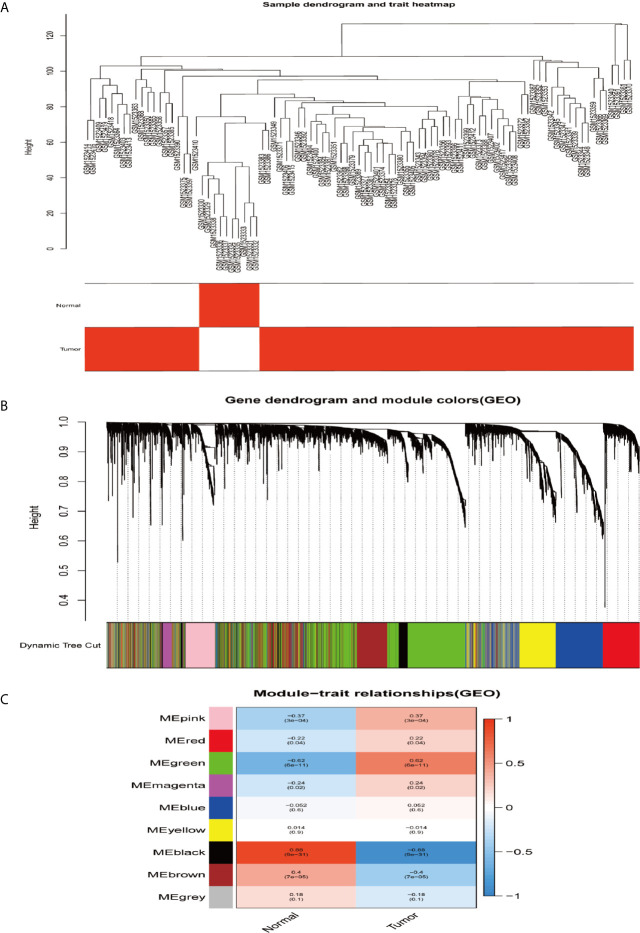
Co-expression modules construction and selection in the GSE62232 dataset. **(A)** Samples clustering and trait heatmap. **(B)** Dendrogram of co-expression network modules were clustered with dissimilarity according to topological overlap (1-TOM). **(C)** Heatmap of the correlation between the module eigengenes (ME) and traits.

**Figure 5:**
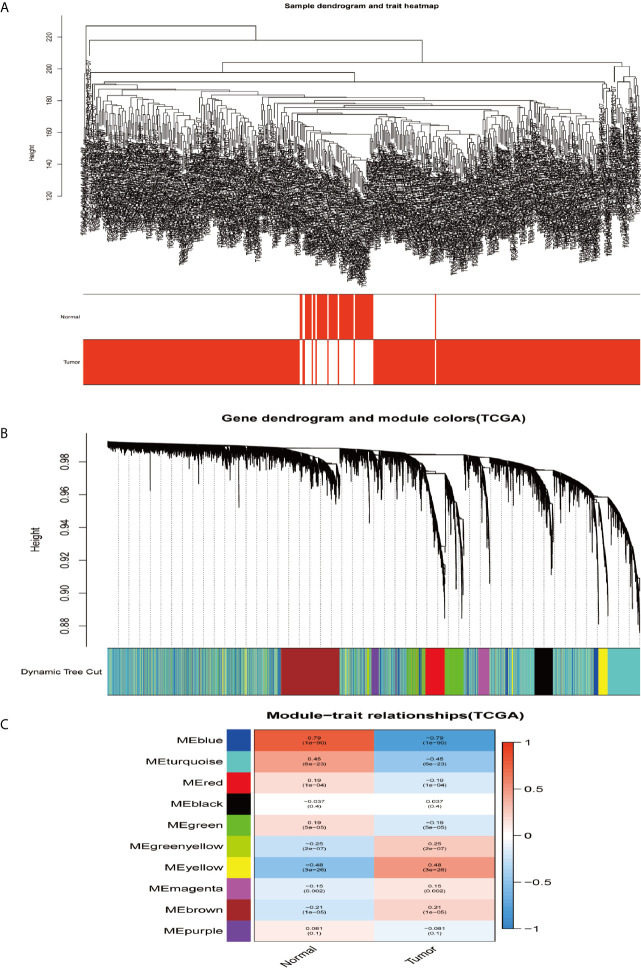
Co-expression modules construction and selection in the TCGA dataset. **(A)** Samples clustering and trait heatmap. **(B)** Dendrogram of co-expression network modules were clustered with dissimilarity according to topological overlap (1-TOM). **(C)** Heatmap of the correlation between the module eigengenes (ME) and traits.

In order to construct the co-expression modules and find the key modules related to HCC, the datasets of GSE62232 and TCGA-HCC were evaluated by the WGCNA package. Hierarchical clustering analyses are shown in **Figures 4A** and **5A**. After assigning colors to each module, a total of nine modules in the GSE62232 (**Figure 4B**), and 10 modules (**Figure 5B**) in TCGA-HCC were identified. Next, we tried to assess the relationship between modules and clinical traits (tumor and normal). The results of the module–trait relationships are presented in **Figures 4C** and **5C**, revealing that the black module in the GSE62232 and the blue module in the TCGA-HCC were found to have the highest association with normal tissues (black module: r = 0.88, *p* = 9e−31; blue module: r = 0.79, *p* = 1e−90).

**Figure 6:**
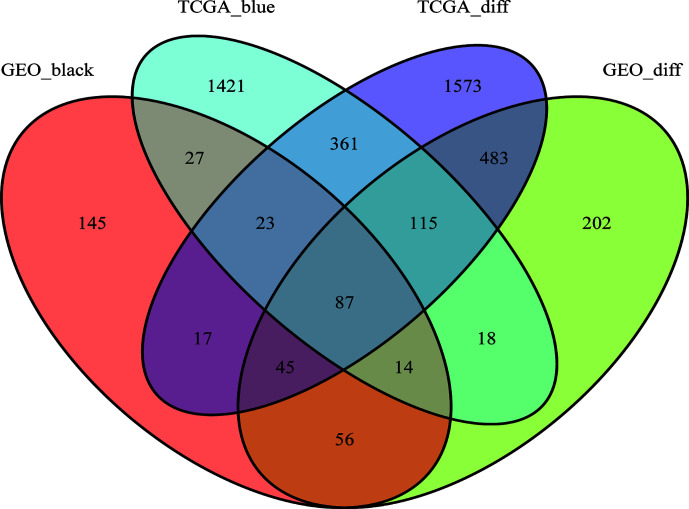
The Venn diagram of genes among DEG lists and co-expression module. In total, 87 overlapping genes in the intersection of DEG lists and two co-expression modules.

As shown in the Venn diagram, 414 and 2,066 co-expression genes were found in the black module of GSE62232 dataset and the blue module of TCGA-HCC, respectively. A total of 87 overlapping genes were extracted to verify the genes of co-expression modules (**Figure 6**).

### Functional Enrichment Analysis of the 87 Overlapping Genes

**Figure 7:**
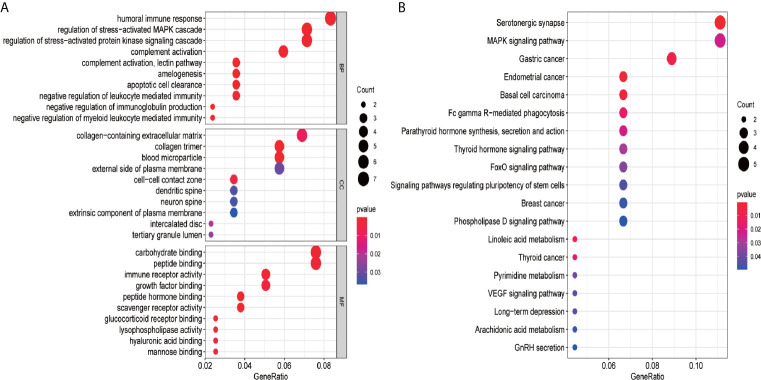
GO and KEGG pathway analysis of the 87 genes. **(A)** GO analysis; **(B)** KEGG pathway analysis. GO analysis includes biological process (BP), cellular component (CC), and molecular function (MF). The count represents the number of genes and the color represents the adjusted p-values.

To gain the potential biological functions and pathway relevance of the 87 genes, GO and KEGG pathway analyses were conducted. The results of GO enrichment analysis showed that the genes were significantly enriched in humoral immune response, collagen-containing extracellular matrix, carbohydrate and peptide binding (**Figure 7A**). Then, KEGG analysis indicated that the genes were mainly enriched in Serotonergic synapse, MAPK signaling pathway and Gastric cancer (**Figure 7B**).

### PPI Network Construction and Hub Genes Screening

**Figure 8:**
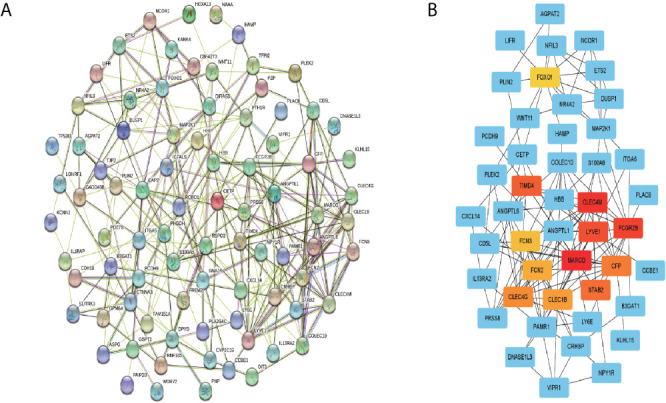
PPI network construction and hub genes screening. **(A)** PPI network of the overlapping genes was constructed using Cytoscape software based on STRING database. **(B)** The top 12 genes with the highest mcc score were selected as hub genes from the PP.

The PPI network of the overlapped genes was constructed with Cytoscape software based on the STRING database, which contains 84 nodes and 269 edges (**Figure 8A**). The hub genes selected from PPI network by MCC algorithm of cytohubba plugin were shown in **Figure 8B**. According to MCC sores, 12 genes with the highest score were selected as the hub genes, including MARCO, CLEC4M, FCGR2B, LYVE1, TIMD4, STAB2, CFP, CLEC4G, CLEC1B, FCN2, FCN3 and FOXO1.

### Validation of the Hub Genes

**Figure 9:**
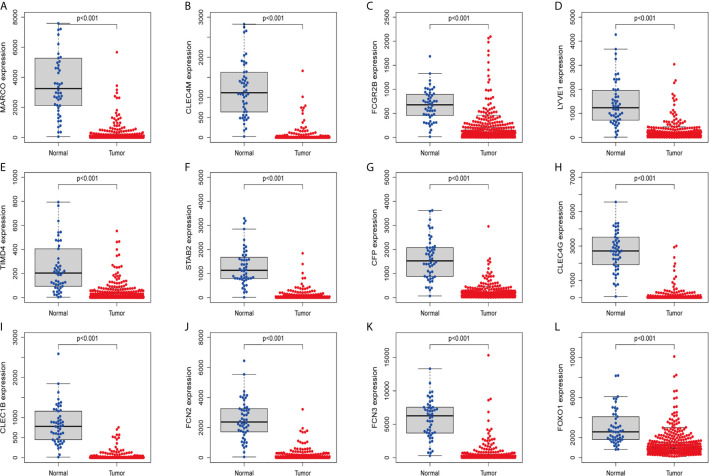
Validation of expression levels of hub genes among HCC and normal tissues from the TCGA database. **(A)** MARCO, **(B)** CLEC4M, **(C)** FCGR2B, **(D)** LYVE1, **(E)** TIMD4, **(F)** STAB2, **(G)** CFP, **(H)** CLEC4G, **(I)** CLEC1B, **(J)** FCN2, **(K)** FCN3 and **(L)** FOXO1.

**Figure 10:**
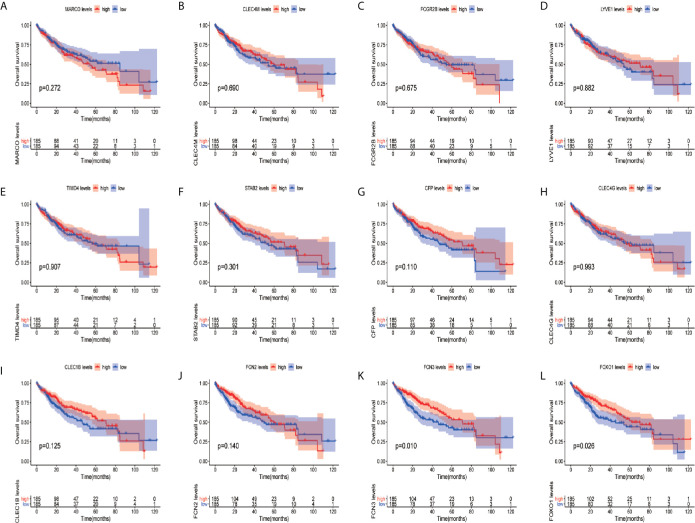
Overall survival (OS) analyses of hub genes. **(A)** MARCO, **(B)** CLEC4M, **(C)** FCGR2B, **(D)** LYVE1, **(E)** TIMD4, **(F)** STAB2, **(G)** CFP, **(H)** CLEC4G, **(I)** CLEC1B, **(J)** FCN2, **(K)** FCN3 and **(L)** FOXO1. The red line represents samples with high gene expression, and the blue line represents samples with low gene expression.

**Figure 11:**
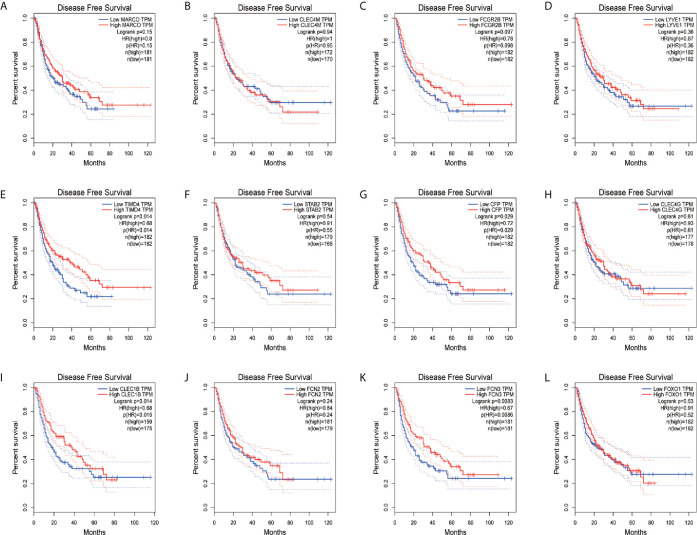
Disease-free survival (DFS) analyses of hub genes. **(A)** MARCO, **(B)** CLEC4M, **(C)** FCGR2B, **(D)** LYVE1, **(E)** TIMD4, **(F)** STAB2, **(G)** CFP, **(H)** CLEC4G, **(I)** CLEC1B, **(J)** FCN2, **(K)** FCN3 and **(L)** FOXO1. The red line represents samples with high gene expression, and the blue line represents samples with low gene expression.

**Figure 12:**
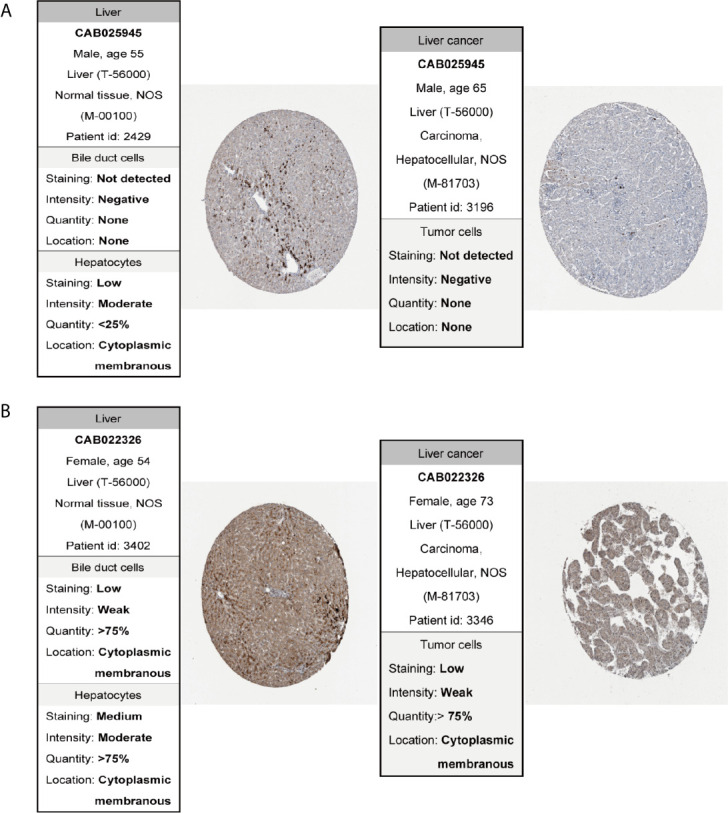
Immunohistochemistry of the genes in HCC (right) and normal tissues (left) from the human protein atlas (HPA) database. **(A)** FCN3, **(B)** FOXO1.

**Figure 13:**
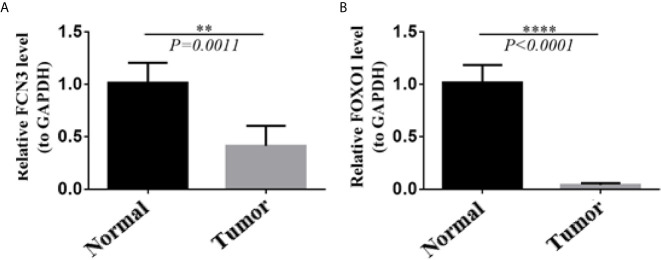
Validation of the expression level of two hub genes using qRT-PCR. **(A)** FCN3, **(B)** FOXO1.

In order to confirm the reliability of the hub genes, we drew a box plot of the expression of each gene between HCC and normal tissues (**Figure 9**). OS analysis of the 12 hub genes were performed by Kaplan–Meier plotter using the R survival package (**Figure 10**). GEPIA2 is an online database, designed to quickly obtain customizable functions. Using this tool, disease-free survival (DFS) curves (**Figure 11**) were obtained and log rank *P* values of 12 hub genes were measured. Among these 12 genes, we found that the expression levels of FCN3 and FOXO1 were significantly related with OS of the HCC patients (*P <*0.05) (**Figures 10K, L**), while with DFS there was no significant difference observed in HCC patients with an expression level of FOXO1 (**Figure 11L**). Moreover, both the immunohistochemical (IHC) staining obtained from the Human Protein Atlas (HPA) database and the qRT-PCR showed a significantly lower expression of FCN3 and FOXO1 in HCC tissues than in normal tissues (**Figures 12** and **13**).

## Discussion

In the clinical application of HCC, traditional diagnostic methods such as serum alpha-fetoprotein (AFP) has limited specificity and sensitivity, current data show that there is no single biomarker used for the detection of HCC alone, especially in the early stages of development (21). Therefore, better biomarkers for specific prognosis and progression of HCC are demanded. Several genes have been identified as novel biomarkers for HCC diagnose. For instance, SCAMP3 was over-expressed in numerous tumors, especially in HCC. SCAMP3 level was positively correlated with disease stages and tumor grades and negatively correlated with patient survival (2); SOX4, STK39, TARBP1, and TDRKH can be regarded as potential prognosticators and therapeutic targets for HCC (22).

In this study, we first combined DEGs and WGCNA to improve the ability to identify genes related to HCC. Our bioinformatics analysis based on TCGA and GEO databases. A total of 87 overlapping genes were extracted to verify the genes of co-expression modules. Based on the Cytoscape software of string database, we consider the top 12 genes as core genes, including: MARCO, CLEC4M, FCGR2B, LYVE1, TIMD4, STAB2, CFP, CLEC4G, CLEC1B, FCN2, FCN3 and FOXO1. Among them, the low expression of FCN3 and foxo1 was significantly related to the OS rate of HCC patients.

FCN3 (Ficolin-3) is a member of fibrin gelatins family, which is mainly expressed in lung and liver. FCN3 can recognize carbohydrate, mediate apoptosis, regulate phagocytosis and activate complement lectin pathway, and participate in local and systemic innate immune response. FCN3 can recognize and bind acetyl compounds and glycosyl structures on the surface of host cells and pathogens, further combine with serine protease, produce living enzymes, hydrolyze complement C4, C2 and C3, form membrane attack complex and initiate cell apoptosis through a series of linkage reactions. The activation of complement lectin pathway mediated by FCN3 is the main defense mechanism of human innate immunity. Studies have shown that the abnormal expression of FCN3 in esophageal cancer, ovarian cancer and other tumors may participate in the host cancer immune response process (23, 24). Previous studies have also shown that FCN3 was overexpressed in the serum of most HCC patients after RFA. FCN3 might be a biomarker for RFA treatment efficacy and a potential target for HCC immunotherapy (25). Our research also shows FCN3 can be regarded as potential prognosticator for HCC.

FOXO1 (Forkhead box O1), also known as the forkhead rhabdomyosarcoma transcription factor (FKHR), is a key member of Forkhead Box family (26). FOXO1 is a factor that inhibits tumorigenesis. Consistently, interruption of FOXO1 level/activity promotes canceration. PI3K, mitogen-activated protein kinase (MAPK) and IκB kinase (IKK) and other major signaling pathways promote carcinogenesis through FOXO family members (27). Studies have shown that low levels of FOXO1 are closely related to digestive system neoplasms (28, 29). Lou et al. found that microRNA-142-5p overexpression inhibits cell growth and induces apoptosis by regulating FOXO1 in Hepatocellular Carcinoma Cells (30). Wang et al. performed the differential co-expression analysis of hepatic gene expression in samples of HCV-cirrhotic patients with and without HCC. They found that FOXO1 might play crucial roles in HCC development (31).

In summary, we provided a comprehensive bioinformatics analysis to identify potential predictive biomarkers between HCC and normal tissues, the present study suggests that the low expression of FCN3 and FOXO1 was significantly related to the OS rate of HCC patients. Our findings provide novel insights into the role of potential biomarkers in HCC and suggest that these findings may have a great clinical significance. However, our article also has many limitations. Firstly, the expression and risk prediction ability of hub genes have not been verified in a large number of clinical samples (22). Secondly, the specific functions of the hub genes in HCC were still missing, we still need to perform experiments to explore this in the future. In addition, it may not be very accurate for each patient with HCC subtypes and we still need to perform experiments to explore this in the future (32).

## Data Availability Statement

The datasets presented in this study can be found in online repositories. The names of the repository/repositories and accession number(s) can be found in the article/supplementary material.

## Author Contributions

SW, ZS, and JinZ designed the study. SW, BT and ZS acquired and analyzed the data. SW, BT and JinZ drafted the manuscript. JianZ and SL reviewed and supervised the work. All authors contributed to the article and approved the submitted version.

## Funding

Our research was supported by the key research fund project of health industry in Tianjin, China (Grant no. 15KG114).

## Conflict of Interest

The authors declare that the research was conducted in the absence of any commercial or financial relationships that could be construed as a potential conflict of interest.
